# Spirit of the British and American Periodicals

**Published:** 1841-10-01

**Authors:** 


### Spirit of the British and American Periodicals &c.

---

THE RETROSPECT OF PRACTICAL MEDICINE AND SURGERY. For the First Half Year of 1841. By W. BRAITHWAITE, M.R.C.S., &c. &c.

WE have, on a previous occasion, expressed a high opinion of Mr. Braithwaite's work. It is one so eminently calculated to be useful, that we hope it will be widely patronised. We shall ourselves notice several of its articles.

1. *The Tinctura Ferri Sesquichloridi in Discharges of Blood from the Urethra.*

Mr. Clay, of Manchester, has contributed some observations on this subject to the Lancet. He says :—In all cases where great discharges of blood have taken place by the urethra, it is generally remarked that the quantity of urine is small; and this is a strong presumptive proof that the disease is seated in the secreting organs, and not in the bladder: under such circumstances we can hope for no advantage by local applications to the inner surface of the bladder; it must be evident, our only advantage is by constitutional treatment, assisted by local applications over the region of the kidneys. In a case that he relates, Mr. Clay prescribed with the happiest effect:—

*Tincture of muriate of iron,* ʒj.; *tincture of opium,* ʒiss.; *infusion of Iceland moss; infusion of gentian,* of each ℥iv. Let an ounce be taken every four hours.

Mr. Clay adds :—Occasionally I have obtained relief from blisters and tartar emetic plaster over the region of the kidneys; but, except in recent cases, and of no great extent, I have not found them of permanent benefit; on the contrary, few cases have withstood the tinct. fer. mur. The number of cases I have seen relieved and cured by it, warrants the conclusion, that the disease is generally in the kidneys, and must therefore be attacked through the constitution.

2. *Stimulating Treatment of Cerebral Disease.*

Mr. E. Copeman has had a paper in the Medical Gazette which contains the following conclusions :—

1. That apoplectic and paralytic affections may take place in an extreme degree without organic disease of the brain.
2. That they often occur from other causes than pressure on the brain.
3. That bleeding, so far from being always necessary, is in many instances prejudicial.
4. That the effort of vomiting is not so prejudicial in these diseases as is generally supposed.
5. That counter-irritation, both external and internal, is a valuable means of affording relief to the symptoms immediately succeeding the attack.

Mr. Copeman inquires, whether there are symptoms generally present in cases of apoplexy and paralysis, which, by strict observation, may enable us to distinguish clearly between those which require bleeding, and those which would be better treated without it. I frankly confess my inability to answer it satisfactorily, and find that others have also failed in doing so. If I may be allowed to give an opinion, I should say that bleeding is unnecessary or prejudicial where the patient is 60 years of age or upwards; where the pulse is feeble, very frequent, intermitting, slow, or large, and inclined to double beat, (I have always found a pulse with double beat indicative of a state of system best relieved by diffusible stimuli,) where the respiration is laboured and accompanied with *cold* perspiration; where there is great mobility of the nervous system with weak muscles, whether the body be thin or *corpulent;* when the attack comes on soon after a full meal, or after great bodily or mental fatigue.

A quick, wiry, resisting pulse, flushed countenance, warm perspiration, noisy breathing, and a tendency to spasmodic muscular contraction, occurring in persons of an earlier age, seem to point out a necessity for resorting to abstraction of blood; but I believe there will be less danger in not bleeding in any case, than in always having recourse to it, where there are *some* circumstances indicative of the propriety of its employment.

### 3. *Guaiacum for Cynanche, and for Rheumatism.*

Several cases of cynanche are related by Mr. Bell, of Banhead, in which half drachm doses of the powdered guaiacum every four or six hours, either alone or mixed up in a suitable form, were attended with very remarkable success; and we recommend the reader to turn to the several cases related in the original paper in the Medical Gazette. Mr. Bell's formula is as follows:

℞ Pulv. Guaiac. ʒiij.; Mucil. G. Arab.; Syrup. Simpl. āā. ℥ij.; Aq. Cinnamom., Aq. Puræ, āā ℥iv. M. et solve. Capt. ℥ij. 4ta. q.q. h.

[One or two days are only required for the cure. In other cases the powdered drug was given in some simpler vehicle. It will be found chiefly successful, if given before suppuration has taken place.]

Guaiacum is a favourite remedy of Dr. Seymour's in rheumatism. In one of his late clinical lectures, at St. George's Hospital, speaking of the treatment of rheumatism, he says:

I generally premise a good bleeding, and afterwards administer the *guaiacum mixture*, and in a few days the patients are well. In the cases at present under our notice, I have followed out this plan, and you have seen how very successful it has proved. How the *guaiacum mixture* acts in these cases I do not pretend to say, except that it causes a profuse increase of the secretions from the skin, the bowels and the bladder; I never find that it begins to effect a cure until all these are fully established.

### 4. *Piperine in Intermittent Fevers.*

Dr. Hartle, Port of Spain, West Indies, writes—I began, as soon as the sweating stage was established, by giving three grains of piperine every hour, until eighteen grains had been taken; and on the following day, when the intermission was complete, I gave the same quantity every three hours. It has in every case succeeded in checking the paroxysm, and as soon as that was accomplished, I gave for some days the following pills:

℞. *Pilulæ Hydrarg.* gr. i.; *Piperinæ*, gr. ij.; *Sulph. Quinæ*, gr. ij.; *Syrup com. q. s. f. M. f. Pil. no.* 1. *omni mane, meridie, et vespere capienda.*

### 5. *Poultices in Inflammations of the Great Cavities.*

The application of poultices in cases of inflammation of the great cavities is a common practice in France. The usual material, says Sir C. Smith, in the Dublin Journal, for poultices is the linseed meal; and they are usually included within linen cloths. The poultices are generally so large as to cover the seat of inflammation. They should be removed and renewed every three or four hours. Particular attention to the latter circumstance is desirable, as the edges are liable to grow cold, and a disagreeable and dangerous dissipation of animal temperature is the result; and in this and in their weight consist, I think, the principal disadvantages of the system.

In the bronchial irritation attendant upon the exanthematous fevers, poultices to the chest are universally employed; and where any want of energy appears to exist in the system, preventing the coming out of the eruption, cataplasms, consisting of the usual linseed meal, with the addition of a very small quantity, say a twelfth or a fifteenth part of mustard meal, (not flour,) are applied to the feet, and maintained for days together; thus keeping up both the temperature of the extremities, and gently stimulating the skin. The same mixture is also, when necessary, applied to the calves of the legs and thighs—the practice in the latter instances being similar in intention to that adopted in Great Britain; but from the mustard meal being less irritating than the flour, and being used in so small a proportion, the sinapized cataplasms can be longer retained in their situation.

Pleurodynia, diaphragmitis, and many other affections of a painful nature, are likewise treated by poultices; and it is certain, that their effects are often, in such cases, very satisfactory.

We have long been in the habit of employing poultices in cases of inflammation of the great cavities. They are certainly useful, but to nothing like the extent which our neighbours of France seem to think. This is indeed a part of their do-little practice, a branch of the tree which bears the sloppy fruit of demi-lavements, ptisans, and so forth. It is chiefly in cases of inflammation of the mucous membranes towards its *decline*, of irritation of those membranes, and of rheumatism, that they are useful. But poultices are not to be a substitute for active treatment—only a rider to it.

6. *Salivation from Iodine and Salivation from Mercury.*

Sir F. Smith writes in the Dublin Journal of Medical Science:—

In the July number of this Journal* I made mention of a case in which I had met with salivation from the use of hydriodate of potass. The fact of salivation occurring from the use of iodine or its preparations is not new: I have, since the publication of the article referred to, met with it in two instances, in one that of a young gentleman using the hydriodate of potass, for a scrofulous necrosis in the bones of the legs; the other that of a young lady, who, for a glandular disease of the neck, was put under the influence of iodine, administered in the watery solution as recommended by Dr. Osborne, of Dublin, at the rate of a grain to the pint; both had been under the influence of the mineral during nearly four months, and had been copiously salivated, and required mercurial purgations, and the administration of bark for the relief of the ptyalism. In each of these cases the entire absence of fetor of the breath was remarked, as was mentioned to have been the case in that which was recorded in the July number. Now, it is worthy of inquiry, why, when fetor is so constantly found to attend salivation by mercury, none should accompany salivation by iodine or its salts? I believe the difference to depend upon the fact that in the action of mercury upon the mouth, there is not only an action induced upon the salivary glands, but also upon the mucous membrane of the mouth, from the alteration in the structure of which latter tissue arises the fetor. If the mucous membrane of the lips, cheeks, or gums be examined with a powerful lens, at the period when mercurial action is commencing in them, a species of ulcerative absorption will be observed to be in progress, which, being increased by the continuance of the cause, soon ends in ulcers of greater or lesser size, perceptible to the naked eye. On the contrary, in the salivation by iodine, the principal, if not the only action of the medicine, is directed to the salivary glands; and though I have in one case observed, with great surprise, the teeth to be loosened, I have never been able to discover any thing like ulcerative action to exist in the mucous membrane of the mouth, and upon this peculiar difference in their relative actions depends, I believe, the presence or absence of fetor of the breath during salivation induced by mercury, and iodine or its compounds.

7. *Pure Tannin for profuse Perspiration.*

Sweating, says Dr. Chervet, is a morbid symptom which is often so serious and inconvenient, that the practitioner is obliged to combat it by special remedies.

---

* The Dublin Journal of Medical Science.

The acetate of lead, which has been extolled of late years, sometimes causes inconveniences which hinder many practitioners from employing it in cases where its use seems clearly indicated. Pure tannin, employed as an anti-sudorific, appears to be free from these disadvantages. The author has employed it for two years at the hospital, and in his private practice, and though it has not succeeded in every case, it has in almost all.

He prescribes it in the form of pill, and in the dose of from two and a half to ten centigrammes (half a grain to two grains) in twenty-four hours, generally in the evening, with or without opium, which neither checks nor favours its action.

8. *Pitch for Piles.*

Dr. Wardleworth assures us that he has used this remedy with advantage in many cases, both of external and internal piles. His usual formula consists in ordering 3½ grains of pitch to be made into pills, two of which are to be taken every evening. The well-known efficacy of balsamic remedies in piles may perhaps serve to recommend these pitch-pills.

9. *Burnt Rhubarb in Diarrhœa.*

Mr. Hoblyn, of the Middlesex Hospital, informs us that—

It may be useful to the profession to know the value of burnt rhubarb in diarrhœa. I have used it for seven years, and found it more serviceable in the diarrhœa, attendant on the last stage of consumption, than the chalk-mixture and opium, or any other of the usual remedies.

I have known it used, with the same pleasing effects, for more than twenty years, in incidental diarrhœas. After one or two doses, the pains quickly subside, and the bowels return to their natural state. The dose is from five to ten grains.

The manner of preparing it is to burn the rhubarb powder in an iron crucible, stirring it until it is blackened; then smother it in a covered jar.

It loses two-thirds of its weight by the incineration. It is nearly tasteless. In no one case where I have known it given has it failed. I have given it in port wine, milk, and water.

10. *Peculiar mode of giving Mercury in Chronic Hydrocephalus.*

Dr. Watson, in his admirable Lectures, tells this case.

An apothecary of considerable experience—now dead—had a lad, fourteen years old, whose name was Scott, under his care, with chronic hydrocephalus, in the year 1817. He had been ill two or three years. He was nearly blind, had very little use of his lower extremities, and could not walk across the room without support. He suffered violent pains in his head, and was unable to bear the least pressure on his scalp. His bowels were constipated, and his pulse "oppressed." Cupping and blistering, and the blue pill, and drastic purgatives, and ordinary diuretics, tried in combination and succession, gave him temporary relief; but no permanent benefit was obtained. Dr. Gower then suggested a plan which he had himself found successful in such cases, and which had first been used by Dr. Carmichael Smyth, who had recorded ten cases of recovery under its adoption. Dr. Gower's plan was to rub down ten grains of crude mercury with about a scruple of manna, and five grains of *fresh* squills: this was to be one dose; and it was to be repeated every eight hours.

My informant rubbed the quicksilver down with conserve of roses, and then added the fresh squills, making the whole into the consistence proper for pills, with liquorice powder. The patient took this dose three times a day, for nearly three weeks, without any ptyalism being produced. Its effects were great prostration of strength, and loss of flesh, with gradual relief of all the boy's sufferings. It operated profusely by the kidneys. The medicine was continued twice a day, and at length once, for another fortnight; when all the symptoms of the disease had disappeared. The boy was greatly emaciated: he was then ordered an ounce and a half of Griffiths' mixture thrice daily; and soon regained his health and strength, and got quite well. And he remained well eight years afterwards.

The same apothecary had another case under his care, which, under the same treatment, terminated equally well.

### 11. *Dangers of Opium in Delirium Tremens.*

Dr. Ware, of Boston, publishes a table of 69 cases of dilirium tremens, treated in different ways.

| Treatment. | Number of Cases. | Bled | Died | Recovered. | Complicated with acute Disease. |
|---|---|---|---|---|---|
| Opium, large doses | 8 | | 4 | 4 | 1 |
| „ small.... | 7 | 1 | 2 | 5 | 1 |
| Emetics ........ | 12 | 1 | 1 | 11 | 2 |
| Bleeding ...... . | 2 | 2 | | 2 | |
| Eclectic ........ | 9 | 5 | 3 | 6 | 7 |
| Quinine ........ | 1 | | | 1 | 1 |
| Mercurials ...... | 1 | | | 1 | |
| Expectant ....... | 29 | 4 | 1 | 28 | 1 |
| | 69 | 13 | 11 | 58 | 13 |

It appears, that of fifteen cases in which opium constituted the principal remedy, six died; whilst of fifty-four in which opium was not used at all, or only incidentally in small quantities, only five died. Still further, if we separate from these fifty-four, the nine cases in which the treatment was eclectic, and in which the mortality seems to have arisen from the combination of acute disease, we have a remainder of forty-five cases, of which only two were fatal.—Again, if we compare the mortality of those cases in which opium was pushed to the full extent advised by writers on this disease, with those in which no active remedy was employed, we have a mortality of one in two, against a mortality of only one in twenty-nine.

This difference in the results of treatment would seem altogether too great to be attributed to accident, and goes far to establish the truth of the opinion formerly expressed, that opium given in large doses is actually injurious to patients labouring under delirium tremens. But even admitting it to be possible, that the great proportion of fatal cases occurring where opium was used, was accidental, it certainly, I think, will not be contended, that the favourable termination of the cases not treated by opium, was also owing to accident. And it will certainly follow that opium, if not actually injurious to these patients, is at least useless, and that our success in this disease will be sufficiently satisfactory without it.

It is certainly difficult to argue against figures, but we are tempted, notwithstanding, to doubt these conclusions. We recollect what delirium tremens was before the opium practice was in common use, and what it is at present, and we can scarcely bring our minds to believe that that practice is not a great improvement. We do conceive that the change of practice has succeeded to a great extent. But those who prefer the old plan, who would rather—

Stare super antiquas vias, may appeal to Dr. Ware's paper.

12. *New kind of Catheter.*

Mr. Foulkes, of Liverpool, has brought this forward. It is to obviate the necessity of using different-sized instruments in cases of spasmodic or permanent stricture of the urethra: or rather to prevent the necessity of withdrawing the one first used when too large, and substituting smaller ones. The instruments are all made of flexible gum. The outer or largest catheter or case contains a smaller one; both these are tipped with silver—within these there is a still smaller one, rounded as a common catheter with an aperture at the side for the escape of urine: they all slide one within the other. The smaller one can be advanced through the contracted part, if the larger one is prevented passing. It is something the same as if we passed a catheter down to the stricture, and when not able to pass it further we advanced the stilette of the catheter through an opening at the end of the catheter and through the stricture. The whole, when put together, forms a sufficiently round point to protect the urethra from injury.

13. *Subcutaneous Division of Bursæ Mucosæ.*

[Professor Williams, of Dublin, has cured several cases of chronic tumours of the bursæ mucosæ and synovial sheaths of tendons "by the subcutaneous division of the sac of the tumour and the subsequent application of pressure."]

A cataract needle was passed into the tumour (an enlarged bursa over the knee), and the entire thickness of the sac divided by several parallel and longitudinal incisions. A portion of the fluid escaped into the cellular tissue, but the greater part found exit externally on the withdrawal of the needle. The knee was then strapped with adhesive plaster, and the limb kept at rest.

[Dr. Houston confirms Mr. Williams's statement by a similar case of his own, respecting which he says,]

The operation was performed with the cataract needle, but as the needle would not have extended much more than half the diameter of the tumour, it was necessary to introduce it first at one side, and then at the other. In this way a number of radiating incisions were made through the sac on both sides, so that there were at least half a dozen incisions on both sides, radiating from the point where the needle was introduced. Next day the tumour had nearly disappeared, and there was no subsequent pain or inflammation. About a tea-spoonful of fluid escaped during the operation.

14. *Excision of Tendons for Paralysis of Muscles.*

Mr. Braid, of Manchester, relates, in the Medical Gazette, several cases of talipes, arising from a *paralytic state* of certain muscles instead of the more common cause, viz., a too powerful or contracted state. For the cure of these cases, he excises a small portion of the particular tendons implicated in the disease, and by the consequent union of the divided ends, he produces such a degree of contraction in the paralytic limb as to act most favourably. Take a case.

Miss ——, æt. six years, has been deprived of the use of the left leg by a paralytic stroke. She has undergone a variety of treatment under some of the first medical gentlemen of this town; but, for the last two years, has been given up as a hopeless case. Her present condition is this: the left leg is perfectly powerless, dangling by the side of her crutch, without reaching the ground, and is much colder than is natural; the foot assuming the appearance of a slight degree of varus, so that it would, if brought to the ground, rest on its outer edge, the toes inclined a little inwards, and the heel slightly elevated. On the 30th of June, 1840, assisted by Mr. Rhind, my son, and one of my apprentices, Mr. Pacey, I made a longitudinal incision along the course of a peronæus tertius, which I elevated, and excised a portion of it, to the extent of three-sixteenths of an inch. I then closed the wound with plaster, and applied splints and bandages, so as to approximate the divided ends, and maintain them in contact.

10th.—In the presence of my talented friend, Dr. J. L. Bardsley, and others, she actually walked across the room and back again without any assistance whatever,—a fact which equally astonished and delighted him and all present. In twenty days she was enabled to walk about in a laced-up boot; and in a week more to throw her crutches aside entirely. She has continued well and strong ever since. Besides the operation nothing else was done in this case, excepting the use of a stimulating liniment, which she had employed before without the least benefit.

15. *Nitrate of Silver for the Stoppage of Hæmorrhage after Extraction of Teeth.*

Mr. Ray has published a case in the Lancet. Upon placing the patient, with a slight inclination backwards, she immediately choked, and it being night, and in the upper jaw near the throat, I had much trouble in obtaining a view of the situation of the hæmorrhage, which flowed as freely, with the pressure of my finger in the socket, as with the addition of small pledgets of lint, steeped in a saturated solution of alum. After an ineffectual trial of these means, I took a stick of nitrate of silver, rounded at the extremity, and firmly forcing it to the bottom of the cavity, allowed it to remain a few seconds in this situation; very little pain was expressed, and, upon its withdrawal, I was gratified in finding a complete cessation of the hæmorrhage. The mouth was kept open with a cork, for about half an hour, to admit air freely, and ensure the preservation of the clot, which might have been disturbed or removed by suction; she then took forty minims of opium and a teaspoonful of compound spirit of ammonia, and afterwards rested five or six hours. With the exception of an unpleasant *cuprous* flavour communicated to all nourishment for the next few days, nothing was complained of but extreme exhaustion, which was many months in disappearing.

16. *Sir B. Brodie on the Treatment of Ununited Fracture.*

Some years ago he had made some experiments on the mode in which fractured bones united, and he had come to the same conclusion as Dupuytren, without having been aware that he had been pursuing the same inquiry. The result of these experiments had satisfied him that union at first depended more upon the neighbouring cellular tissue than upon the periosteum, whatever part the latter might afterwards play in the process. He might take this opportunity of remarking, that he had found the most successful plan of treating ununited fracture, to be that pursued by Mr. Amesbury, and consisting in the binding of the limb in a immovable apparatus, by which the extremities of the fractured bone were kept in a state of forcible apposition for a long period. The plan was a very painful, but a very successful one. He had succeeded in procuring union, by this mode of proceeding, in one case, in which the fracture had been ununited for two or three years, and a seton had been passed through it without benefit, the arm remaining dangling and useless. In the experiments he had alluded to, only one case of union by ligament occurred, and this was in a fracture of the femur of a rabbit. He had endeavoured to account for this deviation from the general rule, but had not succeeded.

17. *The late Mr. Earle on the Mode of Preventing Contraction after Burns.*

I am quite ready to admit that it is not in our power to arrest the law of nature, by which a cicatrized surface becomes smaller, and occupies less space, than the original wound: but it is in our power, in most cases, to direct and modify that which we cannot wholly prevent; and thus, at all events, to counteract its injurious effect...... To take the upper extremity as an example, I will suppose a case, where the whole integuments on the inner and front part of the arm and fore-arm have been destroyed. If such extremity be kept carefully extended on a splint, *not only during the whole process of healing, but long subsequent to the perfect cicatrization,* you will find that the cicatrized surface will diminish in a circular direction, drawing the healthy integuments together from side to side, but that no contraction will take place in the long axis, in which alone it can impede the due motions of the limb. This permanent extension should be persevered in during the *day and night*, until all changes have ceased, and the cicatrix has contracted to its smallest dimensions. Care, however, should be taken, during this time, to give passive motion to the different joints, by which the proper secretion of synovia will be kept up, and the eventual free use of the limb will be ensured. This plan of maintaining the limb in a state of permanent extension should be commenced as soon as the wound has begun to granulate.

18. *Another Infallible Specific for Gonorrhœa.*

M. Gaudriot concludes that the chloride of zinc, properly diluted, has a remarkable power in curing simple gonorrhœa of the urethra and vagina, also in dilating the urethra, and thereby ameliorating strictures. He believes that it is always by an inflammation *sui generis* that the discharge is cured, and that injections of the chloride of zinc determine a series of phenomena not produced by other caustics. It is only necessary to use a few drops of injection, as the extremity of the urethra is the only part to which it should be applied. To combat vaginal gonorrhœa in women M. Gaudriot proposes a new proceeding, which consists in the use of solid vaginal suppositories, composed of a paste made to melt easily, and containing a certain quantity of liquid chloride of zinc and sulphate of morphine.

In men the author employs the following formula:

Liquid chloride of zinc, 24 to 36 drops.
Distilled water, four ounces.
Agitate and filter through paper.

A small quantity of this solution should be injected about an inch along the urethra, two or three times a day.

The vaginal suppository which he employs is formed of—

Liquid chloride of zinc, 5 drops.
Sulphate of morphine half a grain.
Mix with three drachms of the following paste.
Mucilage of gum tragacanth, 6 parts.
Powdered sugar.. .. .. .. 3 „
Starch powder .. .. .. .. 9 „

To cure radically a gonorrhœa in man, it will ordinarily suffice to apply two injections a day for two or three days. The first injections are almost always followed by more or less swelling of the glans penis, but this does not prevent their continuance.

In women four or six suppositories, one being introduced every day, or every second day, suffice to obtain a cure. The first introduction frequently occasions a swelling with more or less heat of the vulva, but these phenomena are soon completely dissipated. Emollient baths have in most of the cases been employed for this purpose.

Those who are easy of faith will believe M. Gaudriot.

19. *The Steam Apparatus.*

A very ingenious one has been invented by M. Duval. The apparatus in appearance is somewhat elegant, is comprised in so small a space that it may readily be carried in the hand, and may be applied either locally or generally, as circumstances may require, with equal facility. It consists of a reservoir for the water, capable of containing a little more than a pint, supported upon three metallic rods, and having a coverlid which is furnished with two openings, one at the centre and one towards the side. From that in the centre arises a tube, terminating in a hollow globe, having attached to it, and communicating with its interior, three short branches furnished with moveable lids. A similar branch is connected with the opening at the upper edge of the reservoir. Beneath, on a pan supporting the parts already described, is placed a spirit lamp, having four burners, and these, when lighted, quickly vaporize the water in the reservoir above. The steam is then conducted to the globe, and thence, by means of short pipes slightly curved, and which may be connected with any one or all of its branches at pleasure, to the part of the body required. The force and the quantity of vapour expelled, is regulated by a key at the side of the principal cylinder, and which will diminish or enlarge its diameter much on the principle of the ordinary stopcock, while its escape upwards is entirely and instantly prevented by exposing the opening at the edge of the reservoir. The way in which it has been used for affections of the joints is simply this—the patient covers the wrist, for instance, with a piece of flannel, large enough for its edges to fall on a pillow, which is placed to support the fore-arm. The nozzle of one of the tubes is then placed beneath the funnel, and the steam allowed to escape. The joint thus enveloped in steam, has usually been allowed to remain for about half an hour; the application being made once a day, or oftener, as the circumstances require. Various modifications of the tubes for different purposes are capable of being adjusted to the openings in the reservoir; but the above-mentioned forms have been found sufficient for such cases as those about to be noticed.

The vapour bath in this form has been introduced into Guy's Hospital within the last two months, and has since been used extensively, and with considerable benefit.

The apparatus is thus used:—The patient lies supine in bed, and three or four slender arches of wood, or other convenient material, are placed across the body, so as effectually to raise the blanket from any contact with it. The apparatus is supported on a stool at the foot of the bed, and one of the pipes allowed to project into the arched cavity, which soon becomes filled with the vapour. In this way all the inconveniences attending a removal to and from the bed are of course got rid of.

### 20. *How to cure Choking.*

Choking, says Dr. Marshall Hall, is only another form of the croup-like convulsion, arising from a more sudden but less permanent *cause*, upon *other* incident excitor *nerves;* and it is to be treated upon precisely similar principles: *the cause must be effectually removed, and as speedily as possible.*

I was witness to a case of choking the other day; respiration was arrested; there was a fearful struggle for breath; not a moment was to be lost; I took the boy and placed him between my knees, so that I could make free pressure on the abdomen, whilst I placed one open hand on the posterior part of the thorax, and struck with the flat part of the other, gently closed, on the anterior, briskly, forcibly; the latter part of this operation was promptly performed, and the little patient was instantly relieved.

### 21. *Suture of the Labia for Procidentia Uteri.*

Professor Geddings has operated successfully in four cases; and the following is the manner in which the operation was performed. The patient was placed in the ordinary position for lithotomy, and the prolapsus reduced. One labium was then put on the stretch by an assistant, and an incision was commenced, with a common scalpel, about a finger's breadth from the upper commissure, and the same distance from the edge of the labium. The incision was carried downwards with a bold sweep, and terminated by a slight curve inwards, and at a little distance behind the fourchette. A slip of the labium of a finger's breadth in thickness was thus severed from the external parts, taking care not to cut through the mucous membrane of the vagina. Making traction on this slip, downwards and inwards, the mucous membrane of the lateral portion of the vagina was then dissected up to the extent of an inch and a half, and detached with the excised labium. The same thing was repeated on the opposite side, the incision being so directed as to intersect the first cut at an acute angle, and remove the fourchette with the other parts. After the hæmorrhage, which was generally inconsiderable, had ceased, an oiled sponge was passed into the vagina, and the two raw surfaces brought into apposition by means of the quilled suture of five stitches. The parts were dressed with a compress of soft lint, secured by a T bandage, and the patient put to bed, with her ankles and knees properly secured together by means of bandages. It was not found necessary to use the catheter, but considerable advantage was derived from keeping the dressings constantly wet with cold water. The introduction of the sponge is for the purpose of preventing the descent of the uterus till adhesion had taken place, and was generally removed about the fifth or sixth day. The sutures were removed in proportion as the parts appeared to be united.

In the four cases operated on by Professor Geddings, no return of the complaint had occurred at the period of the publication of this paper, and they had all returned to their usual avocations.

### 22. *How Artificial Light impairs Vision.*

Dr. Hunter, Surgeon to the Eye-Dispensary, Edinburgh, thus explains it.

Common day-light, which is the only natural light, and the least injurious to the eyes, consists of red, yellow and blue rays in the ratio of 5, 3, and eight; that is a predominance of blue rays, which form the mildest and least hurtful to the retina. In the ordinary forms of artificial light, as the light from candles, either of tallow or wax, oil or coal gas, this proportion is reversed, the yellow and the red rays being in excess, and the blue rays in the ratio of minority. From this peculiarity in artificial light in the excess of red and yellow rays, various inconveniences and evils result.

1. Colours and coloured objects appear in it differently from what they do by day-light. 2. The retina is unequally, we would say too strongly excited, and becomes less sensible to those rays that are in excess, so that afterwards, when it views a white object by day-light, the blue rays contained in the white light reflected from its surface, make a greater impression than the red or yellow rays, and the objects assume more or less of a dingy blue or purple colour. This over-excitement of the retina produces films, *muscæ volitantes*, and even amaurosis. 3. The red and yellow rays possess considerably higher heating powers than the blue rays, and therefore artificial light, which contains these calorific rays in larger proportion, must be injurious to the retina in this manner. 4. From artificial light furnishing less defining power, it is requisite to use it in considerable quantity; and this is another source of over-excitement to the retina. 5. While, in the case of natural or day-light, the ordinary calorific or heating powers of the red and yellow rays are, by various circumstances and beautiful provisions in its transmission through the atmosphere, almost annihilated, so that these rays arrive at the eye with much less of their heating powers than they would have, if direct, the rays of artificial light, by coming more directly and in a more concentrated form on the eye, are accompanied with their concomitant heat, and thereby injure the retina greatly by this hurtful adjunct. 6. The carbonic acid gas which is extricated from all artificial lights, procured as they are by combustion, though not acting on the eye itself, acts on the brain, and especially the optic nerves, the sensibility of which it weakens and ultimately destroys. 7. All artificial lights are unsteady and irregular, and very often placed in improper positions.

Of all artificial lights, gas light is the most hurtful, not from anything peculiarly noxious in its qualities, but from its cheapness, which leads to its being used in excess, and consequently, in such quantity as to over-stimulate the eyes.

To obviate these several evils, Dr. Hunter recommends the more sparing use of gas-light and other artificial lights, and the employment of means calculated either to add blue rays when deficient, or to withdraw the red and yellow rays when in excess.

This object is to be attained by the use of conical reflectors of a bluish or azure colour, and chimneys stained of a blue colour. Pale blue glasses are also recommended; but Dr. Hunter thinks their use is not advisable, as they become hot and require, from the loss of light they cause, to be often removed.

### 23. *True and False Corpora Lutea.*

Dr. Paterson has shewn, that false corpora lutea are frequently connected with menstruation, and has given several cases in illustration. He comes to the following conclusions :—

"False corpora lutea in the human female, arise,

*First,* From the bursting and subsequent filling of a vesicle with blood, as in menstruation.

*Second,* From partial effusion of blood into a vesicle, either with or without rupture of it.

*Third,* By re-absorption of the fluid of a morbidly enlarged Graafian vesicle, giving rise to a puckered cyst.

*Fourth,* From effusion of blood into the tissue of the ovary—the apoplexy of that organ.

*Fifth,* Tubercular deposits.

*Sixth,* Cysts filled with yellow fatty matter.

These are to be distinguished from the true corpus luteum by the following marks —

They in general have an irregular form. They want the central cavity lined with a distinct membrane, or the central puckered cicatrix.

They have no concentric radii.

They are frequently numerous in both ovaries."

---

### Medicine in Egypt.

Dr. Sargent has published in the Dublin Journal* an interesting account of the state of medicine in ancient and in modern Egypt. We shall make a *mem* or two.

*Consecration of Parts of the Body to the Deities.*—The head was consecrated to Phré, the sun; the eyes, to Hathor or Venus; the left temple, to the living spirit of the sun; the nose and lips, to Anubis; the neck, to Isis; and arms, to Osiris; the genitals to Osiris and the goddess Kopt, &c. &c.

*The Moxa in the 16th Century.*—They formed a pyramid of cotton wound round with a silken thread, the base was firmly fixed to the skin, and whilst the combustion was taking place, the surrounding parts were preserved from the effects of radiation; the pyramid was made either solid or hollow to modify the eschar. I have been thus particular to show the minute coincidence of their practice with our, I may say recently introduced, mode of applying the same remedy. The diseases, too, in which it was applied are precisely identical with those in which it is now deemed useful.

*Diseases of Egypt.*—The temperature, the simoom, an insufficient supply of food, its frequently noxious quality, and a degree of filthiness of habit beyond all description, place the mass of the population under physical conditions per-

---

* September, 1841.

haps more disposing to disease than those surrounding any other people. Under these circumstances we cease to wonder at the relation of the numerous severe affections occurring in Egypt. Ophthalmia, in many and obstinate forms, is all but universal; skin diseases of peculiar malignancy and inveteracy abound; variola has been very fatal in Egypt; putrid fevers and intermittents are frequent; phrenitis of great violence also occurs, so as even to destroy life in a few hours; mania, dysentery, and rheumatism are prevalent, along with several surgical disorders, such as calculus, hydrocele, herniæ, &c.; and, to crown the whole, plague in its worst and most destructive forms. Although the older writers mention severe inflammatory affections of the chest as common in the country, Clot Bey asserts, that Egypt enjoys an immunity from those diseases, as also from consumption; and he even proposes a residence there for patients suspected of phthisis.

To meet this mass of disease the native physicians possess neither the elementary principles of their art, nor even the practical skill of former ages; and the best account of Egyptian practice contains merely a record of gross and frequently disgusting superstitions.

*Clot Bey's Introduction of Anatomy into Egypt.*—"I summoned to the proposed seminary the best informed of the rising generation, and 100 young Arabs were the first pupils; new difficulties now presented themselves; we were mutually ignorant of each other's language; how then, I said, were the pupils to be instructed? I fortunately succeeded in finding in Paris three persons who understood French, Italian, and Arabic; but these persons were ignorant of medical knowledge. I told them they should become physicians, but that they should first be pupils. I accordingly set them at work on the translating a treatise on anatomy, lessons from which were dictated to the pupils, who were afterwards examined by means of the interpreters; but plates and wax models having failed to communicate the necessary anatomical knowledge, the dissection of the actual subject became essential: but here almost insuperable obstacles were before us, independently of the idea of profanation of the body. The Egyptians have a theological doctrine that the dead feel the tortures to which they may be subjected; the Pacha and the Minister of War refused to undertake the responsibility of sanctioning the practice; at length, however, the chief of the Ulemas, a learned and enlightened man, was induced to *connive* at it; two pupils were induced to begin on detached portions of the body, they were soon cured of their prejudices, and convinced of the indispensability of dissection; in turn they convinced their parents and relatives, who convinced the rest of the people; at length, from mere toleration, we were afforded actual encouragement, and finally, *Ibrahim Pacha and his Ministers of State assisted at a lecture on practical anatomy.*"

Whatever M. Clot's faults may be, and his advocacy of his patron has been carried to a reprehensible extent, he is unquestionably a man of genius.

---

### MR. HAMILTON ON PARTIAL RUPTURE OF NERVES.*

After relating some cases, Mr. Hamilton observes:—In these cases the cause of the injury was pretty nearly similar, the part having been put violently on the stretch; a sense of something having broken was followed by pain, referred to the anatomical position of a nerve, but besides this, parts supplied by branches of the nerve are affected with paralysis of sense and motion, leaving diagnosis of

---

* Dublin Journal, September.

injury of the trunk of the nerve extremely probable. Another symptom of uncommon interest in Essy M'Mahon's case is the alteration in the temperature of the hand. The extreme coldness observed in this case I have seen in many others where a nerve had been injured, and is mentioned by Sir B. Brodie as among the symptoms of local hysterical effections of the joints. It is not merely the sensation of cold experienced by the patient himself, but an absolute diminution of temperature, attended with a mottled bluish appearance, and sometimes an œdematous tumefaction of the part. The coldness is usually not persistent, but alternates with intense heat, which last sensation is infinitely more distressing than that of the cold. The hot alternation appears occasionally to obey certain laws, coming on at two periods in the day as hectic does, viz. about two or three o'clock in the day, and again at bed time. The part is flushed, and sometimes a perpiration breaks out in it, and it literally smokes, the rest of the body being natural.

---

### Benjamin Franklin's Estimate of Animal Magnetism.

Franklin thus writes to M. De La Condamine.

You desire my sentiments concerning the cures performed by Camus and Mesmer. I think, that, in general, maladies caused by obstructions, may be treated by electricity with advantage. As to the animal magnetism, so much talked of, I must doubt its existence till I can see or feel some effect of it. None of the cures said to be performed by it have fallen under my observation, and there being so many disorders which cure themselves, and such a disposition in mankind to deceive themselves and one another, on these occasions, and living long, has given me so frequent opportunities of seeing certain remedies cried up as curing every thing, and yet soon after totally laid aside as useless, I cannot but fear that the expectation of great advantage from this new method of treating diseases will prove a delusion. That delusion may, however, in some cases be of use while it lasts. There are in every great, rich city, a number of persons, who are never in health, because they are fond of medicines, and always taking them, whereby they derange the natural functions, and hurt their constitution. If these people can be persuaded to forbear these drugs, in expectation of being cured by only the physician's finger, or an iron rod pointing at them, they may possibly find good effects, though they may mistake the cause.*

I have the honour to be, &c. B. Franklin.

—*Dublin Med. Press,* July 21, 1841.

---

### Spurious Opium.

Mr. Mason, at the Meeting of the Pharmaceutical Society, on August 11th, made

---

* In writing to Dr. Ingenhousz, some time afterwards on this subject. Dr. Franklin said, "Mesmer is still here and has still some adherents and some practice. It is surprising how much credulity still subsists in the world. I suppose all the physicians in France put together, have not made so much money, during the time he has been here, as he alone has done. And we have now a fresh folly. A magnetiser pretends, that he can, by establishing what is called a *rapport* between any person and a *somnambule,* put it in the power of that person to direct the actions of the *somnumbule,* by a simple strong volition only, without speaking or making any signs; and many people daily flock to see this strange operation."—*Spark's Life of Franklin.*

a report on a sample of spurious opium, which had lately been offered to the trade by a person calling himself a drug broker. It had been sent to him only a few hours ago by Mr. Lescher, and from the experiments which he had made upon it, he had not been able to detect any appreciable quantity of morphia, and but a trace of meconic acid. He thought it possible that it might be a mixture of a very small proportion of bad opium with the refuse of a morphia manufactory. He intended to prosecute his experiments further, but had fully satisfied himself of its entire worthlessness. Mr. Morson hoped that the circumstance would serve as a sufficient example of the impropriety of purchasing drugs of this description from strangers, since those who do so have no reason to complain of any imposition which may be practised upon them.

The spurious opium, Mr. Jacob Bell informs us, was in globular masses covered with leaves, had rather the appearance of Constantinople or Smyrna opium; when cut it was not unlike ordinary dark coloured opium, and had a slight liquorice odour. It appears to become moist on exposure to the air.*

This enables us to introduce to our readers the Transactions of the Pharmaceutical Society, an excellent institution, in its infancy at present, but promising great things for pharmacy. We wish it every success.

---

### MR. FERRALL ON THE ANATOMY OF CERTAIN PARTS WITHIN THE ORBIT.

Mr. Ferrall writes a long paper on this matter.

1. *Anatomy of the Superior Eye-lid—its Continuity with Fibrous Tissues within the Orbit.*

Having placed, says Mr. Ferrall, the eyelid on the stretch, and fixed it in this position by chain hooks, an incision should be made through the integuments, commencing at the superciliary ridge, and extending vertically downwards to the ciliary margin of the lid. In examining the successive layers, those at one side only of the line of incision should be raised, and followed into the orbit; the other half of the eyelid should be left untouched, in order to preserve a view of the section itself afterwards. Having raised the common integuments, and orbicularis muscle, with which every anatomist is familiar, the part which now presents itself is a distinct layer of fascia. When this is followed carefully upwards, it will be found to pass into the orbit, and is the first element of the eyelid which can be traced into that cavity. Within the orbit, this layer corresponds superiorly to the lachrymal gland—the vessels—and the fat which has been described as pervading every portion of the cavity, and to be in contact even with the globe of the eye. Beneath this layer, however, or among any of the tissues between it and the eyeball, no adipose substance is to be found.

The next part which presents itself in this dissection is, the levator palpebræ muscle. The mode of insertion of this muscle, although exceedingly interesting, does not properly belong to the present inquiry. Beneath the levator palpebræ another fascia, similar to the former, is met with. This also passes into the orbit, and uniting at the edges of the levator, with that already described, seems to constitute a perfect sheath for its accommodation and support.

The part next in order is the tarsal cartilage. Tracing this upwards and backwards, we find that its thin or orbital margin is continuous with another fibrous lamina of greater strength and more remarkable appearance than those already alluded to. Following it into the orbit, it will be found to separate the supe-

---

* Pharmaceutical Transactions, edited by Jacob Bell, No. III.

rior rectus muscle from the globe of the eye, but presenting a well-defined opening, through which the tendon of the muscle passes as over a pulley, to be inserted into the sclerotic coat. The external or orbitar aspect of this fibrous organ is cellular, and will scarcely prepare the anatomist for the very different appearance which it presents on its ocular surface, or that in contact with the globe of the eye. This inspection must, however, be made from the front or corneal aspect of the orbit.

2. *Anatomy of the Tunica Vaginalis Oculi, and Muscles of the Eye.*

As was before remarked, the inspection must be made from the front or corneal aspect of the eye. The following is the mode which I adopt. I divide both palpebræ vertically, and turning the separated portions backwards towards the forehead and cheek respectively, fix them in this position by hooks; the conjunctiva is next divided at its angle of reflection, where it passes from the internal surface of the eyelid to the ball of the eye. The incision being made, and the edges of the divided membrane separated, the anatomist is at once struck with the inaccuracy of all former descriptions of the parts. Zinn, Soemmering, and all former anatomists, describe the ball of the eye as being cushioned on fat, and in immediate contact with its muscles. Mr. Lawrence, no inconsiderable authority on this subject, and whose opinion may be supposed to represent that of all living anatomists, speaking of the anatomy of the conjunctiva, says, "thus, this membrane is reflected from the palpebræ to the whole circumference of the globe, forming a circular fold, which, at the point of reflection, corresponds to the fat of the orbit."

Having, however, separated the divided conjunctiva, we expose, not as has beed described by anatomists, a cushion of adipose tissue, but a distinct tunic of a yellowish white colour, and fibrous consistence, continuous in front with the posterior margin of the tarsal cartilage, and extending backwards to the bottom or apex of the orbit, where its consistence becomes less strongly marked. By proceeding in the manner I have mentioned, the parts are displayed without any elaborate dissection. The sharp end of a probe, or director, will be sufficient to separate the ball of the eye from the new organ, by breaking gently the fine cellular tissue which connects them. Its colour is totally different from that belonging to its external surface, and it is here perfectly smooth, where the eye glides over it in its movements. The muscular substance of the recti muscles is no where visible, they lie on the outside of this tunic, which insulates and protects the eye in the most perfect manner.

The most beautiful portion of this mechanism, however, remains to be described. In the concavity of this tunic, and about half an inch posterior to its anterior or orbitar margin, are to be found six well-defined openings, through which the tendons of the muscles emerge in passing to their insertion in the sclerotic coat, and over which they play, as over pulleys, in their course. The tendons are loosely connected to the edges of those apertures by fine cellular tissue, which opposes no obstacle to their gliding movements.

Mr. Ferrall attributes two important offices to this tunica vaginalis oculi—it invests and protects the globe of the eye, and it regulates the direction in which the muscles of the organ shall exert their force. It especially, according to him, prevents retraction of the eye.

3. *Inflammation of the Tunica Vaginalis Oculi.*

In common with fibrous tissues in other situations, this tunic is subject to inflammation and its consequences. It appears also to suffer from similar constitutional causes, and to present analogous phenomena, when it becomes the seat of disease. This may be combined with other affections of the orbit; and it may be the primary affection, and the point of departure from which the diseased action may spread to the other fibrous layers in the orbit, and finally reach the periosteum; and the attack may even be limited to the tunica vaginalis oculi —that it may here produce a train of symptoms of the most dangerous kind, and which have been hitherto supposed to reside in the periosteum, because the existence of other fibrous membranes in the cavity was not suspected. Presuming that there were no other tissues in the orbit, to which to attribute the disease, practitioners naturally referred the majority of cases to one or other of those with which they were acquainted. The solution of such cases would have been less difficult, if our clinical researches were based upon a more correct knowledge of the structures actually existing in the orbit.

Mr. Ferrall relates two cases, for which we refer to our contemporary.

Mr. Ferrall quotes the following passage from M'Kenzie, to tack to it an ingenious commentary.

" 'It sometimes happens,' says Mr. M'Kenzie, in his valuable work on Diseases of the Eye, 'that the cellular membrane of the orbit does not suppurate. On the subsidence of the acute symptoms, the eyeball, in such cases, is found to be deprived of its power of motion, is protruded, or has even become amaurotic from the pressure it has undergone.'

From what I have observed, I am induced to believe, that the real cause of the motionless state of the eyeball in such cases, is adhesion of the globe to the tunica vaginalis, as well as consolidation of the several other fibrous layers which envelop it, and form the thecæ of the muscles. Adhesion of the tunica to the ball of the eye will, it may be supposed, be accompanied by abnormal union of the tendons of the muscles to the edges of the openings through which they pass in the natural state. The rotation of the eye upon its axis will, of course, be impeded in proportion to the interruption to their usual gliding movements."

4. *Abscess between the Tunic and Globe of the Eye.*

When matter is formed in this situation, it may be distinguished from an abscess external to the fibrous tissues, by its pointing in the angle between the ball of the eye and lid. In this case the fold is obliterated, and the conjunctiva is rounded into the form of a tumour.

5. *Tumours within the Tunica Vaginalis Oculi.*

It will often require a good deal of discrimination to decide on the most eligible situation for making the incision for the removal of the tumours from the cavity of the orbit. The displacement of the eye, and the projection of the palpebra, will not always suggest the exact locality of the tumour. The eyelid may be thrown forward in such a manner as to make the practitioner suppose that the morbid growth lay nearer to the orbit than the eyeball, and very little covered by soft parts. If a careful examination be not premised, the external appearances may mislead him, and he may find that his incisions have to pass through a great depth of parts, to remove a tumour which actually lay in contact with the eyeball. Tumours may, in fact, form internal or external to the capsule of the eye, and the mode of operating should be regulated by an exact knowledge of their locality with respect to this tuuic.

6. *Extirpation of the Globe.*

When the disease is confined to the globe of the eye, and does not involve the optic nerve or the orbital appendages, Mr. Ferrall suggests the following operation.

The conjunctiva being freely divided, the six tendons may be snipped across with a scissors one after another, where they emerge from the tunic. The eyeball will then be easily detached by a probe or director passed freely around it; when one step alone would remain—the division of the optic nerve. When we recollect that the roof of the orbit is occasionally found to be as thin as paper in some parts, it will appear most desirable to avoid stripping it of its coverings, by operating within this second orbit, or proper fibrous socket of the eye.

---

### Case of Retraction of the Testicle to the Abdominal Ring.

Dr. Murphy, of Liverpool, relates the following case.

August 20, 1838. A foreign gentlemen, æt. 30, was suddenly seized with the following symptoms: an excruciating pain in the right groin, increased on the slightest motion of the corresponding lower extremity; a tumour larger than a marble, and sensible to the slightest pressure, is found at the external ring; no impulse is communicated to it by coughing; the testis is absent from the scrotum, a circumstance before noticed.

He is subject to this attack five or six times in the year; the pain continues from ten minutes to an hour, and then suddenly subsides, leaving him perfectly well. When the pain is more severe than usual there is vomiting.

A large dose of laudanum was prescribed, and an exhausted cupping-glass applied over the tumour. The pain ceased in a few minutes, after having continued more than twenty. When the cupping-glass was removed he walked about; the testicle had descended, and the chord, which was larger and softer than usual, felt as if it was varicose, but could not be lessened by pressure either upwards or downwards. A very eminent surgeon considered it hernia; but he had never seen him during the attack.

My impression, says Dr. Murphy, of the disease was, that the testicle was attached to the omentum, by which it was occasionally retracted to the orifice of the ring. A nicely fitted truss was applied over the external ring. Fifteen months have now elapsed without a return of pain. I have a distinct recollection of having read of a similar case, and the sufferer, I think, was the celebrated Zimmerman. If the truss failed in warding off these attacks, he gave his consent to permit me to cut down on the chord and divide the omentum, or even the chord, should I deem it necessary.*

---

### Dr. Macartney's Irrigating Apparatus.

To keep a limb, or part, continually wetted, Dr. Macartney has contrived an apparatus expressly designed for irrigation, by means of which, water of any requisite temperature can be employed, and conveyed to the affected part underneath the bedclothes. The apparatus consists of a box made of zinc, something like a fracture-box, in which either the upper or the lower extremity of the patient may be placed. The water, or a medicated fluid, is carried in a flat tube, which at one end is connected with the reservoir, and the other end projects through a slit in the upper edge of the box. The tube contains a strip of coarse woollen cloth, which is broad at one end and pointed at the other. The broad end is received by the vessel that contains the fluid, and the pointed end either rests upon the dressings of the affected part, or is suspended immediately over them. The water is taken up by the strip of cloth, and carried along it on the principle of capillary attraction, or in the manner of a syphon; and thus a continued supply of water is conducted to the part affected, without inconvenience or exposure to the patient. In order to get rid of the fluid, there is a concave bottom, perforated with large holes, through which the fluid passes into the

---

* Dub. Journ. July, 1841.

inferior part of the box, and from which it is conveyed by a tube into any vessel that may be placed outside the bed for the purpose of receiving it. The perforated bottom, for the sake of cleanness, is made to take out; and there is a soft cushion, covered with painted linen, on which the limb rests, and consequently the whole is not kept wet.

The quantity of fluid which may pass can be regulated by placing the vessel or reservoir containing the water either higher, or on the same plane as the patient's bed. If placed high, so much as three gallons of fluid may be supplied in the course of twenty-four hours. If warm water be required, as in cases of strains and lacerated wounds, the temperature of the reservoir may be kept up by means of a spirit lamp.

The irrigating machine is made and sold by Messrs. Weiss and Son, instrument makers, 63, Strand, London.

---

### CROTON OIL IN NERVOUS DISORDERS.

Mr. Cochrane has communicated some cases to the Edinburgh Monthly Journal,* illustrative of the virtues of croton oil. We shall select one.

"In December 1839, I was called to attend a female patient, whom I found in bed completely prostrate, and apparently insensible to every thing around her. In fact she was completely comatose,—her pupils were greatly contracted, her pulse could scarcely be felt, and, in fine, she seemed all but sunk into the sleep of death. The remedy which I employed in this case was the croton oil, in combination with mucilage and castor oil, thus proportioned:

℞. Ol. Tiglii, gtt. viij.
— Ricini, ℥iij.
Mucil. G. Arab. ℥i. Misce bene.
Sign. Enema. To be administered in a quart of gruel.

Though I employed the above potent remedy, I must say, I scarcely expected any good to result from it; but to my great joy and satisfaction, it had a most beneficial effect upon the patient, not by producing an alvine torrent, but by occasioning a very copious discharge from the bowels per anum, as well as from the stomach, by vomiting, together with a return of sensation and motion, and the use of her mental faculties. Indeed, the effect of the medicine was truly astonishing, for when I left her, she seemed all but dead. On my second visit, only a few hours afterwards, she was able to sit up in bed and converse with those around her."

It appears to us that cases of this kind are reported too loosely, to afford as much assistance to practical medicine as they might. Nothing is said of the state of the bowels prior to the administration of the croton oil. Yet it is clear that their condition must exercise a paramount influence on the prospects of advantage from the remedy. Such a state of nervous system, as obtained in the case related, *might* co-exist with relaxation of the bowels, as well as the reverse, with an irritable state, forbidding purgation and purgatives, as well as with one demanding them.

---

### ELATERIUM AND AMMONIATED TARTRATE OF IRON IN CHOREA.†

Mr. Wardleworth thinks that the indications for treatment in chorea are, 1st,

* July, 1841. † Ibid.

to unload the bowels, if a torpid condition of them exists; and, 2dly, to restore the tone of the stomach and intestines. The purgative formula which he has found most efficacious is the following:—

℞. Ext. Elaterii, gr. j.
Pulv. Jalapæ, gr. xxxvj.
— Zingib. gr. xxiv. Misce et divide in chart. xij.
capt. j. quarta quaque horâ donec alvus soluta fuerit.

After the bowels have been fully acted on, he prescribes that important therapeutic agent, the ammoniated tartrate of iron,—a remedy which, though little known, still possesses chalybeate properties far superior to any other preparation of iron, with the exception of the magnetic oxide. Its pleasant taste, and perfect solubility in water, render it exceedingly agreeable even to the most fastidious.

He commences by giving three grains three times a-day (in quovis vehiculo,) the dose to be gradually increased to five grains, which will generally be found sufficient to subdue the disease, care being taken at the same time to keep up a free action of the bowels, combined with a light yet nutritive diet. Free exercise in the open air, after the disease appears to decline, is essential to the patient's perfect recovery. From a fortnight to three weeks is about the average time required by the proposed treatment to subdue the worst forms of chorea.

---

### Treatment of Tropical Dysentery.*

Dr. Lewins, of Leith, has contributed a paper on this subject to our Northern contemporary: the paper will repay perusal. The gist of it—the treatment recommended by the author—we extract.

If, from any circumstances, the symptoms at their outset have been neglected, blood-letting, topical and general, is most imperatively called for. If pain increased on pressure, furred tongue, and other evidences of inflammation exist, no more potent remedial measures can be employed, but if such symptoms be absent, as they will be in the great majority of instances in the early stage, they are unnecessary, and will generally be found to produce no decided immediate relief to the tormina, tenesmus, and diarrhœa, the three most distressing symptoms. After blood-letting, three grains of opium, combined with large doses of calomel, are usually administered, and small opiates and starch injections are frequently employed. Of these, and particularly of the enemata, Dr. L. speaks in the highest terms, having found them to produce much greater relief than the blood-letting. Occasionally it relieved for some hours the urgent symptoms, and even where such success did not attend its employment, and where, from the irritability of the rectum, it was soon discharged, the tenesmus was often considerably mitigated. The introduction of the pipe sometimes however produced irritation, and occasionally forced the patient to stool. The large mucilaginous injections of the Continental School are justly reprobated by our author. Dr. Lewins thinks full purgative doses of sulphate of magnesia, or castor oil, often hazardous and inexpedient in the early, though beneficial in the later stages. The diet should be strictly antiphlogistic; rice water used lukewarm is perhaps the best thing the patient can take. If leeches be applied, either for inflammation in the liver or intestines, the patient should be strictly protected from cold during the operation. The entire class of astringent remedies, so useful in the diarrhœa of Europe, act as irritants, and are wholly inadmissible.

---

* Edinburgh Monthly Journal, July, 1841.

Ipecacuan and various other remedies have been much lauded by men of the greatest experience. Dover's powder is undoubtedly serviceable, particularly if the disease becomes chronic, and the same remarks apply to the terebinthinate liniments. A warm and equable temperature is of great importance in the cure of this affection, and warmth to the belly ought constantly to be applied. This treatment, which was the one I first adopted, proved tolerably successful. The pain was lessened, inflammation warded off, the tenesmus and tormina were relieved, and the stools became less frequent. The quantity of opium and calomel used during the progress of the disease is sometimes very considerable, though it is by no means necessary to induce salivation to effect a cure. During the early part of his convalescence, the patient is very desponding, haggard, hypochondriacal, and very weak. He is very restless, there is great want of tone in the digestive organs, and an aching void is complained of in the abdomen, to relieve which, Battley's sedative liquor at bedtime is a most excellent remedy. It calms the nervous system, procures undisturbed sleep, and acts as a tonic, and is unquestionably to be preferred to any other preparation of opium. The diet should at first be strictly farinaceous. Laxatives are now required, and as castor oil even in small doses sometimes acts violently, and always increases the derangement of stomach, rhubarb is a preferable medicine. The improvement is frequently very slow, the greatest care being requisite to prevent errors of diet, and the abdomen should be well protected by clothing from vicissitudes of temperature. If at this stage there should be signs of subacute inflammation in the abdomen, leeches must be perseveringly applied, and subsequently, as a safeguard, an eruption should be excited by an ointment composed of one ounce of axunge, half a drachm of the tartrate of antimony, and five grains of the bichloride of mercury,—the eruption being kept out for several weeks. In severe cases of dysentery, complete recovery often takes place very slowly; the digestive organs are much weakened, diarrhœa comes on from the slightest causes, and occasionally for months there is a slight discharge of blood along with the stools, which all the remedial measures you may employ are unable to check, and which is at length cured by change of air. The rapid recovery which takes place when the patient is restored to his native climate, is frequently most surprising and gratifying.

But Dr. Lewins praises another method as superior to the foregoing. It was that pursued by Dr. Heyn at Java. His treatment consisted in administering, at the very outset of the disease, a scruple of the chloride of mercury. According to his experience, this powerful pharmaceutical agent, when employed in small doses, acts as an irritant, but when given in large quantities, has so decided and immediate an effect, as to merit the name of a direct sedative. I have seen soldiers brought into hospital in the most severe agony, discharging mucus and blood at very short intervals, and labouring under the most aggravated form of the disease. On administering a scruple dose of calomel, the most marked and gratifying amelioration took place. The tormina, tenesmus, and diarrhœa almost instantaneously cease, a feeling of comfort takes the place of the most intense suffering, the countenance brightens up, the pulse, heat of tongue, and thirst, when present, are diminished, and in short all the distressing symptoms are much alleviated. This remission is, however, often followed by an exacerbation at the end of six or eight hours. Re-action is established, the pulse beginning to rise, and, if no steps are taken to prevent it, the whole train of symptoms return with almost undiminished violence. If the diarrhœa and tenesmus come on, no hesitation need be felt in repeating the scruple dose of calomel, but if the physician be on his guard, he may often prevent this, by active measures whenever the tormina and borborygmus are apparent. This is the critical moment, and a scruple of calomel divided into four doses, administered every three hours, will often prove sufficient to prevent re-action, and overcome the disease. If the malady be obstinate, five grains of calomel should be given boldly every three or four hours, and under this treatment the medical man will seldom be disappointed. When the bowels have been quiet for thirty-six hours, a scruple of the powder of rhubarb was found to be most serviceable. The stools which followed were fetid, dark-coloured, fluid, sometimes bloody, but voided with little or no pain. If symptoms of inflammation appeared in any of the abdominal viscera, which rarely happened, leeches were used; but a blister was almost invariably applied to act as a safeguard, and to relieve the remaining irritation in the intestines. The superiority of this treatment over the other is, he thinks, unquestionable.

---

### Extraction of Foreign Bodies from the Ear by Syringing with Cold Water.*

Mr. Carpenter, of Castlecomer, thus writes to our Dublin contemporary: The first case brought to me, some years back, was one in which the foreign body was a garden pea, which, as is usual, was pushed in as far as the tympanum by the interference of the child's friends. The instrument I selected was a very small forceps with blunt points, with which I could catch the pea, but could not move it, and, on endeavouring to do so, caused intolerable pain. It immediately occurred to me to inject cold water with force, in order to at least displace the pea, and perhaps thereby render it more manageable—I did so with a two ounce syringe, and found the pea so far displaced as to lie at the orifice of the meatus, whence I removed it without further trouble or pain to the child. Since that I have extracted many peas in the same way—in some instances they have lain at the orifice, and in others been forced out completely. So far I was satisfied that peas could be removed by the above method, and will admit that I felt anxious to have an opportunity of trying my hand on something of more difficult extraction. About six months back that offered—the substance was a pebble of a most irregular shape, and so large that I was surprised how the child could have borne to introduce it—the meatus was not inflamed; but the pebble was so far in, and so wedged in its place, that to touch it ever so lightly with any instrument, was almost enough to throw the child into convulsions. I almost despaired of succeeding with the syringe, but made the trial, and not having a two ounce one at hand used a pint one—the first injection failed; but, while using a second, the pebble, much to my satisfaction, was forced out on the napkin held under.

In the last case brought to me I could hardly feel the foreign body with a silver probe, nor could the child's parent tell me what it was; however, knowing it to be small, I used a small sized syringe, and by one injection had a grain of oats at the orifice of the meatus.

---

### Mr. Donovan's improved Method of preparing Vinum Ferri.†

Take of the best hock one pint; common rust of iron of the shops, well levigated, two ounces. Introduce both into a matrass, which plunge into a water bath maintained at the temperature of 100°. Constantly agitate the matrass for an hour; then remove it from the water, and the next day filter. The colour of this vinum ferri is a very deep greenish brown, almost black when the volume is

---

* Dub. Med. Press, June 30, 1841.
† Dub. Med. Press, June 23, 1841.

great: its taste is ferruginous, agreeably and highly vinous; it produces a pleasant warmth in the stomach, and never sickens. In its effects it must be tonic, diuretic, emmenagogue, anthelmintic, and carminative. It does not, in a moderate dose, excite.

No other wine than hock will afford a preparation possessing these virtues. The dose for an adult may be three or four drachms thrice a day; in smaller doses, it is of little use. If it is to be exhibited in combination with a bitter, it agrees well with colombo or gentian.

By this method, in one day, we obtain a far better preparation than is procurable by the processes of the pharmacopœias in two months. The iron exists in it chiefly in the state of protoxide.

---

### ROYAL COLLEGE OF SURGEONS IN LONDON.

The Council proposing to publish, in the course of the ensuing year, a Volume, to be entitled

"*Transactions of the Royal College of Surgeons in London,*"

invite, from the Members of the College and other scientific persons, communications relating to the improvement of anatomical and surgical Science.

The subjects proposed to be included in this Publication are specified in the following extract from the Ordinances of the College:—

"The Transactions shall consist of

Original Communications on Surgical subjects.

Collegial and Jacksonian Prize Dissertations, deemed of sufficient originality and merit.

Original Memoirs on Human Anatomy.

Original Memoirs on Comparative Anatomy.

Anatomical Monographs of rare Animals, dissected in the Museum of the College.

Explanations of, and Commentaries on, important Preparations in the Museum, with illustrative Plates.

Statistical Reports from Hospitals."

It is requested that Papers intended for publication in this Volume may be transmitted to the President, at the College, on or before the 1st of May, 1842.

EDMUND BELFOUR,
Secretary.

28th July, 1841.

